# Fabrication and validation of an LED array microscope for multimodal, quantitative imaging

**DOI:** 10.1016/j.ohx.2023.e00399

**Published:** 2023-01-24

**Authors:** Tarek E. Moustafa, Edward R. Polanco, Rachel L. Belote, Robert L. Judson-Torres, Thomas A. Zangle

**Affiliations:** a Department of Chemical Engineering, University of Utah, Salt Lake City, UT, USA; b Huntsman Cancer Institute, University of Utah, Salt Lake City, UT, USA; c Department of Dermatology, University of Utah, Salt Lake City, UT, USA

**Keywords:** LED array microscope, Multimodal microscopy, Quantitative phase imaging, Darkfield imaging, Calibration and validation

## Abstract

The combination of multiple imaging modalities in a single microscopy system can enable new insights into biological processes. In this work, we describe the construction and rigorous characterization of a custom microscope with multimodal imaging in a single, cost-effective system. Our design utilizes advances in LED technology, robotics, and open-source software, along with existing optical components and precision optomechanical parts to offer a modular and versatile design. This microscope is operated using software written in Arduino and Python and has the ability to run multi-day automated imaging experiments when placed inside of a cell culture incubator. Additionally, we provide and demonstrate methods to validate images taken in brightfield and darkfield, along with validation and optimization for differential phase contrast (DPC) quantitative phase imaging.

## Graphical abstract

**Figure:**
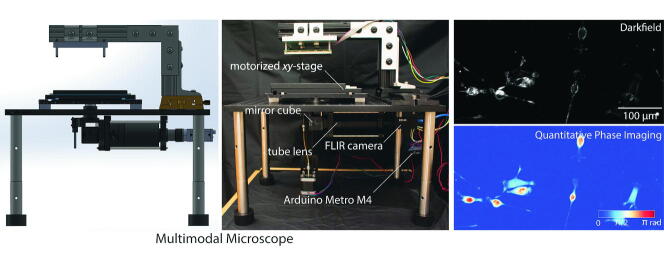

**Specifications table:** 

Hardware name	Multimodal LED array microscope
Subject area	Biological sciences (e.g., microbiology and biochemistry)
Hardware type	Imaging tools
Closest commercial analog	Phasics SID-4 Bio, PHI Holomonitor live cell imaging system
Open source license	CC BY-SA
Cost of hardware	$16,436
Source file repository	https://doi.org/10.5281/zenodo.6841618

## Hardware in context

Live cell imaging is a powerful approach to study and quantify the behavior of individual cells. The development of new microscopy hardware supports this approach by increasing the accessibility and precision of live cell imaging. In particular, the combination of multiple approaches in a multimodal microscope can be used to obtain complementary data about a sample. Many different microscope modalities can be readily combined on a single microscope such as darkfield (DF) and fluorescence microscopy [1] and fluorescence microscopy and quantitative phase imaging (QPI) [2]. An emerging trend in multimodal microscopy is the use of computational methods to capture brightfield, darkfield, phase contrast, and quantitative phase microscopy images using illumination provided by a single LED array [3], [4], [5], [6]. Computational microscopy techniques leverage the versatility of an LED array to either illuminate LEDs within the numerical aperture (NA) of the objective lens to produce brightfield illumination or to illuminate LEDs outside the NA of the objective lens to produce darkfield illumination. Furthermore, LED arrays can be used to produce asymmetric illumination to produce images containing data on the gradient of phase shifts through the sample such that the total phase shift can be retrieved computationally to implement quantitative phase imaging (QPI) [3], [5], [6], [7].

Open-source systems enable researchers to build their own microscopes for a fraction of the cost of a commercial system while also having the flexibility of being customizable for the unique needs of the researcher, such as the ability to add brightfield, darkfield, fluorescence or phase contrast modalities. One downside to many systems incorporating QPI with one or more of these modalities is that they often require complex optical configurations. This is one of the factors behind the emergence of computational microscopy and LED array microscopes [4], [5], [6], [8]. In addition to these open-source hardware configurations, open-source software packages have been developed to facilitate communication between the different components (such as x-y-z stages, illumination, and image acquisition) [9], [10].

When used alone or in addition to other imaging modalities, label free imaging techniques such as QPI offer opportunities to study live single cell attributes like mass accumulation, heterogeneity, and drug response [11]. QPI measures the phase shift as light passes through a transparent sample such as microbeads or live cells. This phase shift, also called the optical path length, is integrated over the area of the sample to find the difference in refractive index between the sample and the surrounding medium. For samples such as cells, the optical volume can be used to compute cell mass [12], [13], and by measuring the mass over time can be used to determine cell growth for studying cell behavior in response to growth perturbations such as cancer therapy [14].

There are numerous examples of commercially available and open-source microscopes and/or microscope attachments that implement QPI along with other microscopy modalities. This can be achieved, for example, by purchasing a commercial research microscope, such as those available from Olympus, Nikon, or Zeiss, and outfitting it with a commercially available phase imaging system from Phasics or Phi Optics. Then, due to the multiple output ports on the microscope, these commercial research microscopes can be readily adapted to combine QPI with other microscopy modalities such as brightfield, phase contrast, fluorescence, or darkfield microscopy depending on the application. In addition to these, Phase Holographic Imaging produces a microscope for measuring the phase shift of live cells, with a small form factor allowing it to fit inside a small tissue culture incubator while imaging, or with two systems inside a standard size incubator. The Livecyte microscope, sold by PhaseFocus, integrates brightfield, QPI, as well as 7 channels of fluorescence imaging with onboard tissue culture conditions for live cell imaging all into a single platform. While several commercial options are available, most are very expensive, often costing upwards of $100,000 for the entire system. Therefore, important alternatives to these commercial systems are open-source platforms that enable researchers to build their own microscopes to substitute for these expensive platforms.

However, an important problem facing researchers that use these open-source systems is the rigorous characterization of the system that is required to validate quantitative measurements. For users of commercial systems, such calibration is often provided or performed by the manufacturer. In terms of user-performed calibrations, previous work in QPI has used expensive phase targets or microbeads in commercially available index matching fluid for quantitative phase validation [15]. However, there is less work describing how to tune QPI measurement and phase retrieval algorithms or how to optimize darkfield illumination for maximum contrast when using custom-built systems.

Here we demonstrate the fabrication and validation of a multimodal LED array microscope. We demonstrate the validation and fidelity of quantitative phase measurements using microbeads embedded in an NOA73 (Norland Products, USA) medium through accurately and reproducibly assessing the difference in refractive index between the two materials. QPI is then used to demonstrate how to tune the measurement and reconstruction parameters of the system by measuring the optical volume of the polystyrene beads, with validation against the theoretical expectation. Furthermore, we demonstrate how to determine the correct illumination angles for darkfield microscopy and characterize the signal-to-noise ratio (SNR) of the system.

## Hardware description

### Microscope overview

**Fig. 1:**
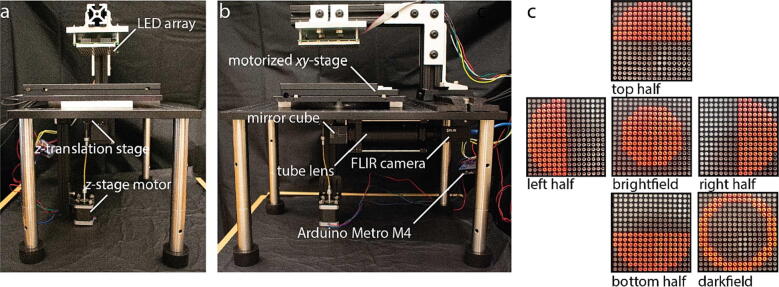
Microscope and illumination patterns. (a) Front view and (b) side view photographs of multimodal LED array microscope. (c) Photographs of LED array illumination patterns for the various microscopy modalities such as brightfield, darkfield, and the asymmetric illumination patterns for QPI.

The multimodal microscope presented here requires two major components for operation, the LED array, along with the required electronics to drive it, and the optical system (Fig. 1a, b). In addition to these major components there are motorized stages to allow movement in the xy plane (the sample plane) and along the z-axis (parallel to the optical path) to facilitate locating the region of interest on the sample for imaging. Finally, the system includes the optomechanical parts required to fix the geometric arrangement of these components relative to each other. The entire system has dimensions of 460 × 380 × 465 mm (depth × width × height) and is sized to fit within a standard 150 L (5.3 cu ft) cell culture incubator.

### LED array

We used a 16x24 LED array with 4.7 mm pitch for sample illumination (Fig. 1a, b). The LED array is a low-cost illumination source that is readily programmed using open-source libraries provided by Adafruit. The LED array is factory mounted to a circuit board that facilitates I2C (inter-integrated circuit) communication with the Arduino used to drive the LED array. The LED array is programmable such that any desired pattern can be displayed on the array for the various microscopy modes. For example, using half circle illumination we can acquire 4 images that can be used to reconstruct the phase shift that occurs as light passes through the sample [4], [5], [6], [7] (Fig. 1c). A single full circle illumination pattern, or the sum of opposing pairs of half circle images (e.g. left + right), can be used to obtain a brightfield image. We can obtain darkfield images by using the LEDs for illumination that are outside the numerical aperture of the objective lens [4] (Fig. 1c).

### Arduino Metro M4 Express

We used an Arduino Metro M4 Express to drive the LED array and the stepper motor used for the Z-stage to focus the microscope. The Arduino Metro M4 Express is a custom Arduino designed by Adafruit built on the open-source Arduino platform and is up to 10x faster than a traditional Arduino Uno with a clock speed of 120 MHz. This faster Arduino microcontroller allows the pattern on the LED array to be changed several times per second. The Arduino Metro M4 is USB compatible, controllable with a computer via a serial connection, and interfaces with devices such as the LED array through the I2C protocol using the open-source libraries provided by Adafruit. The Arduino Metro M4 Express is an inexpensive microcontroller, but can be readily replaced by a Metro M4 Grand Central if more input/output (I/O) pins are needed for niche applications or by an Arduino Uno/Mega if rapidly changing the pattern on the LED array is not required (Fig. 1a,b and Fig. 4).

### Arduino Protoboard

The Arduino Protoboard is used to interface the Arduino Metro M4 express with the other electronic components it is used to control. Pins are soldered into the protoboard so that it can be plugged into the I/O pins on the Arduino Metro M4 Express, connecting them to leads on the protoboard. Wires are then soldered into the protoboard to create permanent connections between the electrical components, such as the motor and LED array, and the I/O pins on the Arduino (Fig. 4).

### Bipolar stepper motor

The bipolar stepper motor (3.9 V) is used for focusing the microscope (Fig. 1a, b). Stepper motors such as these are widely used for many applications and are therefore readily compatible with the Arduino Metro M4 Express using a TI-L293DNE controller. The TI-L293DNE controller is an integrated circuit made by Texas instruments, is readily available, inexpensive, and greatly facilitates wiring and programming the stepper motor to move in precise increments.

### Optical components

**Fig. 2:**
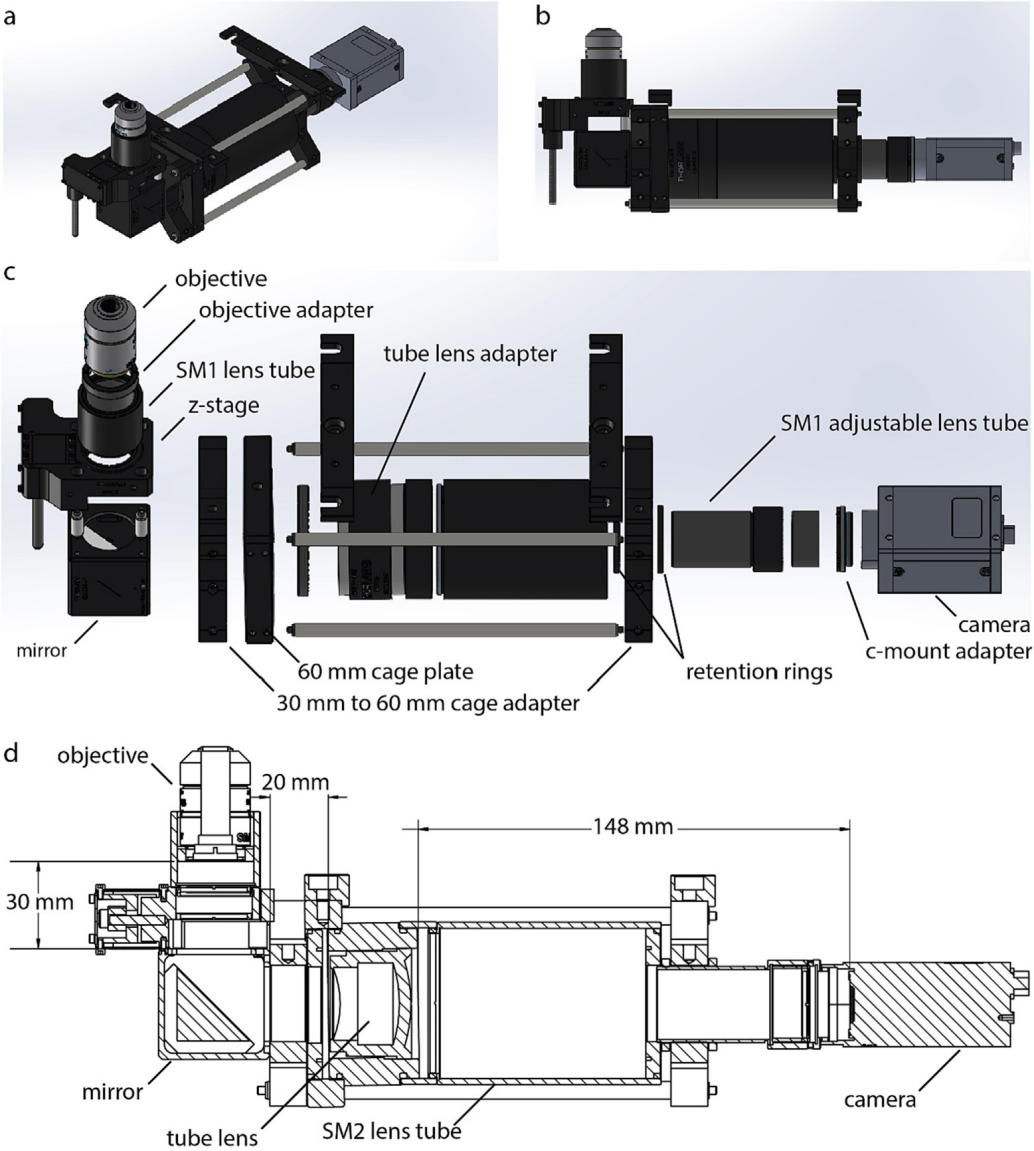
Optical assembly. (a) Isometric view of 3D model of the optical assembly. (b) Side view of 3D model of optical assembly. (c) Exploded view, 45° above side view, of how the optical components and hardware are arranged. (d) Cross-section drawing of the optical path showing the distance between each of the optical components used for image acquisition.

For detection, we used a simple optical system composed of an objective lens, a mirror, a tube lens, and a camera. Isometric and side views of a 3D model show the fully built detection arm separate from the rest of the constructed microscope (Fig. 2a, b). We used a 10x Plan N Olympus objective lens with a numerical aperture (NA) of 0.25 to collect light from the sample. We used a 10x objective because of the large field of view, and the lower NA decreases the distance at which LEDs must sit relative to the sample plane to use the microscope for QPI or darkfield. This is characterized by the coherence parameter, σ:

(1) $$\sigma =\frac{NA_{\mathrm{illumination}}}{NA_{\mathrm{objective}}}$$

Increasing the coherence parameter increases the range of spatial frequencies collected by the system. For obtaining QPI images the coherence parameter must be greater than 1, with a value of approximately 1.5 giving improved image quality [4]. For darkfield, which uses annular illumination, the illumination is defined by two coherence parameters, one each for the inner and outer angles of illumination. The inner coherence parameter is always greater than 1, and the outer coherence parameter is set according to the limits of the LED array, combined with the distance from the sample. Light collected using the objective lens is then focused onto the camera using a 200 mm focal length tube lens making this microscope composed of a simple two lens system.

### GS3-U3-23S6M-C CMOS Mono Camera

We used a Grasshopper3 machine vision camera from Teledyne-FLIR for image acquisition (GS3-U3-23S6M-C). This camera readily interfaces with the SM1 threads of the Thorlabs lens tubes used here for optomechanics using a C-mount adapter. The camera can be used with an SDK provided by Teledyne-FLIR, allowing it to be controlled using Python software to modify settings such as exposure time, frame rate, gain, and bit depth, and used for image acquisition.

### High-speed translation stage

We used a Thorlabs high-speed translation stage for high-speed imaging on this microscope. The stage can be controlled using a joystick for easy use. The Thorlabs XY-stage can also be controlled using Python so that the settings can be changed during long periods of imaging or automated experiments. We chose this stage specifically to match the speed and accuracy requirements of our experiments. However, it can also be swapped for other open hardware designs that significantly lower the overall cost of the system [16], [17], [18].

### Z-translation stage

The objective lens on this microscope is mounted on a manual translation stage connected to a bipolar stepper motor to automate its movements. The stage has a range of 1.5 mm, long enough for most focusing applications. The high resolution of this stage is determined by the combination of the low translation per revolution (50 µm/rev) and the 400 steps per revolution operation of the stepper motor giving a translation of 0.125 µm/step.

## Design files summary

**Table:** 

Design file name	File type	Open source license	Location of the file
AdapterForStageDrawing	.pdf	CC BY-SA 4.0	https://doi.org/10.5281/zenodo.6841618
LEDArrayAdapterDrawing	.pdf	CC BY-SA 4.0	https://doi.org/10.5281/zenodo.6841618
BreadboardDrawing	.pdf	CC BY-SA 4.0	https://doi.org/10.5281/zenodo.6841618
MicroscopePlatformDrawing	.pdf	CC BY-SA 4.0	https://doi.org/10.5281/zenodo.6841618
IlluminationArmDrawing	.pdf	CC BY-SA 4.0	https://doi.org/10.5281/zenodo.6841618
FullMicroscopeAssemblyDrawing	.pdf	CC BY-SA 4.0	https://doi.org/10.5281/zenodo.6841618
DetectionArmDrawing	.pdf	CC BY-SA 4.0	https://doi.org/10.5281/zenodo.6841618
MultiModalMicroscopeSoftware	.zip	CC BY-SA 4.0	https://doi.org/10.5281/zenodo.6841618
AdapterForStageModel	.sldprt	CC BY-SA 4.0	https://doi.org/10.5281/zenodo.6841618
LEDArrayAdapterModel	.sldprt	CC BY-SA 4.0	https://doi.org/10.5281/zenodo.6841618
FullMicroscopeAssembly	.sldasm	CC BY-SA 4.0	https://doi.org/10.5281/zenodo.6841618
IlluminationArmAssembly	.sldasm	CC BY-SA 4.0	https://doi.org/10.5281/zenodo.6841618
MicroscopePlatformAssembly	.sldasm	CC BY-SA 4.0	https://doi.org/10.5281/zenodo.6841618
DetectionArmAssembly	.sldasm	CC BY-SA 4.0	https://doi.org/10.5281/zenodo.6841618

## Bill of materials summary

**Table:** 

**Designator**	**Component**	**Number**	**Cost per unit**	**Total cost**	**Source of materials**	**Material type**
Camera	GS3-U3-23S6M-C 1/1.2″ FLIR Grasshopper®3 High Performance USB 3.0 Monochrome Camera	1	$1,250.00	$1,250.00	https://bit.ly/37jG9bG	Semi-conductor
LED array	16x24 Red LED Matrix Panel - Chainable HT1632C Driver	1	$24.95	$24.95	https://bit.ly/3rt55o7	Semi-conductor
C-mount adapter	SM1A9 - Adapter with External C-Mount Threads and Internal SM1 Threads, 4.4 mm Spacer	1	$19.96	$19.96	https://bit.ly/39dzRM0	Metal
SM1 coupler	SM1T2 - SM1 (1.035″-40) Coupler, External Threads, 0.5″ Long, Two Locking Rings	2	$22.08	$44.16	https://bit.ly/3xlFuQc	Metal
SM1 adjustable lens tube	SM1V10 - Ø1″ Adjustable Lens Tube, 0.81″ Travel Range	1	$34.51	$34.51	https://bit.ly/3xlFuQc	Metal
X-Shaped Adapter	30 mm to 60 mm Cage Plate Adapter, 8–32 Tap	2	$43.26	$86.52	https://bit.ly/37MJRup	Metal
SM2-SM1 adapter	SM2A6 - Adapter with External SM2 Threads and Internal SM1 Threads	1	$26.51	$26.51	https://bit.ly/37mF0jN	Metal
SM2 Lens Tube	SM2L30 - SM2 Lens Tube, 3″ Thread Depth, One Retaining Ring Included	1	$38.09	$38.09	https://bit.ly/3vrW9ke	Metal
Tube Lens	ITL200 - Tube Lens, f = 200 mm, External M38 × 0.5 Threads	1	$509.12	$509.12	https://bit.ly/3OgQc24	Inorganic
Tube lens Adapter	SM2A20 - Adapter with External SM2 Threads and Internal M38 × 0.5 Threads	1	$51.66	$51.66	https://bit.ly/3rsCCih	Metal
SM2 coupler	SM2T1 - SM2 (2.035″-40) Coupler, Internal Threads	1	$20.41	$20.41	https://bit.ly/3mEt4hn	Metal
60 mm Cage Plate	LCP01 − 60 mm Cage Plate, SM2 Threads, 0.5″ Thick, 8–32 Tap	1	$42.15	$42.15	https://bit.ly/3EfO8CG	Metal
8-inch rods	ER8-P4 - Cage Assembly Rod, 8″ Long, Ø6 mm, 4 Pack	1	$45.79	$45.79	https://bit.ly/3EwdyMN	Metal
Half inch rods	Cage Assembly Rod, 1/2″ Long, Ø6 mm, 4 Pack	1	$19.77	$19.77	https://bit.ly/3MaT7HQ	Metal
1-inch rods	Cage Assembly Rod, 1″ Long, Ø6 mm, 4 Pack	1	$19.77	$19.77	https://bit.ly/3EfOtp6	Metal
Z-Stage	Z-Axis Translation Mount, 30 mm Cage Compatible	1	$205.60	$205.60	https://bit.ly/3OaTnrY	Metal
Fine Hex Adjuster	Fine Hex Adjuster, 3/16″-100, 2.00″ Long	1	$9.90	$9.90	https://bit.ly/3jJPkoq	Metal
Motor	Nema 17 Bipolar 0.9deg 36Ncm	1	$14.65	$14.65	https://bit.ly/3XBXd1J	Semi-conductor
Aluminum Breadboard	MB1218 - Aluminum Breadboard 12″ x 18″ x 1/2″, 1/4″-20 Taps	1	$204.43	$204.43	https://bit.ly/3MbMhSl	Metal
Rubber Feet	AV6 - Ø45.0 mm Sorbothane Feet, Internal 1/4″-20 Mounting Thread, 4 Pieces	1	$34.11	$34.11	https://bit.ly/3uJ0yjm	Polymer
Feet posts	RS6 - Ø1″ Pillar Post, 1/4″-20 Taps, L = 6″	4	$35.24	$140.96	https://bit.ly/3L1UZCB	Metal
Optical Rail	Precision Optical Rail, 12.46 in. Length, 3.93 in. Width, 12 in. Scale	1	$251.00	$251.00	https://bit.ly/3KKI6wd	Metal
Rail Carrier	Optical Rail Carrier, 3.0 in. Length, 1/4–20 Thread, PRL Series	1	$115.00	$115.00	https://bit.ly/3JJhVVG	Metal
T-framing fastener	End-Feed Double Nut, Flanged-Button Head 1/4″-20 Thread	2	$5.51	$11.02	https://bit.ly/3xw5wSg	Metal
T-slotted frame mounting foot for 1.5″ rails	Silver Mounting Foot for 1.5″ High Single Rail	1	$22.17	$22.17	https://bit.ly/3zvX4nd	Metal
Motor rail	Single Four Slot Rail, Silver, 1″ High × 1″ Wide, Solid	6	$0.79	$4.74	https://bit.ly/3jKe67S	Metal
Motor Bracket	Nema 17 Bracket for Stepper Motor and Geared Stepper Motor Alloy Steel Bracket	1	$1.50	$1.50	https://bit.ly/3EwGbtf	Metal
Flexible Shaft	Flexible Shaft (rad. of curve = 100 mm)	1	$35.23	$35.23	https://bit.ly/3vg9qfs	Metal
Aluminum Bar	Multipurpose 6061 Aluminum 1/2″ Thick × 2″ Wide 1 ft long	1	$15.10	$15.10	https://bit.ly/3OdJnOJ	Metal
1/4–20 Screws	1″ 1/4 20 Threads screws (Pack of 10)	1	$4.33	$4.33	https://bit.ly/3KP4fcT	Metal
M4 Screws	M4 2.5 cm (Pack of 25)	1	$10.22	$10.22	https://bit.ly/3xrRwJh	Metal
Optical Rail	Precision Optical Rail, 12.46 in. Length, 3.93 in. Width, 12 in. Scale	1	$251.00	$251.00	https://bit.ly/3KKI6wd	Metal
Corner brackets	Silver Slotted Corner Bracket for 1.5″ High Rail, 1.5″ Long	3	$7.27	$21.81	https://bit.ly/3uIax8I	Metal
Corner- brackets	Silver Slotted Corner Bracket for 1.5″ High Rail, 1.5″ Long	4	$8.10	$32.40	https://bit.ly/3Pexcku	Metal
Shims	26-Piece 18–8 Stainless Steel Slotted Shim Set, Trade Size A	1	$25.15	$25.15	https://bit.ly/3zDcAh2	Metal
T-slotted rails	T-slotted framing - Single 4-Slot Rail, Silver, 1.5″ High × 1.5″ Wide, Hollow	2	$9.81	$19.62	https://bit.ly/3NOCcuY	Metal
T-slotted rails	T-slotted framing - Single 4-Slot Rail, Silver, 1″ High × 1″ Wide, Solid	1	$11.04	$11.04	https://bit.ly/3ItyZjb	Metal
Right-angle corner brackets	Silver Flush 90 Degree Angle Bracket for 1.5″ High Rail	2	$19.41	$38.82	https://bit.ly/3tDYTuG	Metal
Aluminum Sheet	Multipurpose 6061 Aluminum 7/16″ Thick × 6″ Wide × 1/2 ft	1	$16.92	$16.92	https://bit.ly/3O7ndgW	Metal
M4 Screws	M4 2.5 cm (Pack of 25)	1	$10.22	$10.22	https://bit.ly/3xrRwJh	Metal
Spacer	Aluminum Unthreaded Spacer 3/16″ OD, 3/4″ Long, for Number 4 Screw Size	6	$0.59	$3.54	https://bit.ly/3M6PLoX	Metal
LED Screws	18–8 Stainless Steel Socket Head Screw 4–40 Thread Size, 1–1/2″ Long, Partially Threaded	1	$13.73	$13.73	https://bit.ly/3vi5M4U	Metal
LED Nuts	18–8 Stainless Steel Hex Nut 4–40 Thread Size	1	$3.89	$3.89	https://bit.ly/3KYUPvm	Metal
Aluminum Bar	Multipurpose 6061 Aluminum 1/2″ Thick × 2″ Wide 1 ft long	1	$15.10	$15.10	https://bit.ly/3OdJnOJ	Metal
XY-Stage	MLS203-1 – Fast XY Scanning Stage	1	$7,719.84	$7,719.84	https://bit.ly/3NFck5u	Metal
Joystick	MJC001 – 2-Axis Microscopy Joystick Console	1	$1,125.81	$1,125.84	https://bit.ly/3xBRxKr	Metal
XY-Stage Controller	BBD302 – 2-Channel Benchtop 3-Phase Brushless DC Servo Controller	1	$3,405.47	$3,405.47	https://bit.ly/3NFG81E	Metal
Metro	Adafruit Metro M4 feat. Microchip ATSAMD51	1	$27.50	$27.50	https://bit.ly/3jJOTdM	Semi-conductor
Protoshield	Adafruit Proto shield for Arduino	1	$9.95	$9.95	https://bit.ly/3vhYuOA	Semi-conductor
Transparent Ruler	1 mm Stage Micrometer with 10 µm Divisions	1	$185.18	$185.18	http://bit.ly/3CpWo3g	Inorganic
TI-L293DNE controller	Texas Instruments L293DNE	1	$3.08	$3.08	https://bit.ly/3CLqpLp	Semi-conductor
Mirror	30 mm Cage Cube-Mounted Protected Silver Turning Mirror	1	$182.02	$182.02	https://bit.ly/3M3ALIA	Inorganic

## Build instructions

### Optical assembly

The isometric and side views of the 3D model are shown to give an overview of the optical assembly isolated from the rest of the microscope platform (Fig. 2a, b). To assemble the optical assembly:

• Begin by screwing the c-mount adapter into the c-mount threads of the camera.
• Attach SM1 coupler without its outer retention rings to the SM1 internal threads on the c-mount adapter.
• Screw the SM1 adjustable lens tube into the SM1 coupler.
• Screw an outer retention ring onto the SM1 adjustable lens tube then screw a 30 mm to 60 mm cage adapter (X-shaped), then another outer retention ring followed by an SM2-SM1 adapter.
• Screw the SM2 lens tube onto the SM2-SM1 adapter till the edge of the lens tube is flush against the surface of the adapter.
• Prepare the tube lens by screwing it into the tube lens adapter.
• Using the SM2 coupler, connect the end of the SM2 lens tube and the image plane side of the tube lens adapter.
• Screw the 60 mm cage plate onto the objective plane side of the tube lens adapter.
• Using a pair of calipers and following Fig. 2c and Fig. 2d, measure the distance between the edge of the tube lens adapter closest to the camera to the casing of the camera. This distance should be 144 mm plus the 4 mm between the casing and the position of the camera sensor inside the camera giving a 148 mm total distance. The measured distance should be within a tolerance of 3 mm which is the tolerance of the camera sensor placement. As needed, adjust the SM1 adjustable lens tube to the correct length and lock it in place using the retention ring. Make sure the X-shaped adapter and 60 mm cage plate both have 60 mm holes in line with each other.
• Slot the 8-inch rods along the 60 mm holes to connect the 60 mm cage plate and the X-shaped adapter to prevent the system from rotating.
• Slide another X-shaped adapter onto the other side of the 60 mm cage plate using the 8-inch rods and lock everything along the 8-inch rods in place by tightening the screws
• Prepare the mirror by screwing in the half inch rods.
• Attach the mirror to the X-shaped adapter by sliding the rails and locking them in place.
• Screw the 1-inch rods into the mirror.
• Prepare the Z-stage by unscrewing the knob until it is completely out, then screw in the fine hex adjuster.
• Screw the objective lens into the objective lens adapter, then screw the group into the Z-stage.
• Slide the Z-stage onto the 1-inch rods.
• Cover the rails between the mirror and Z-stage with black aluminum foil, being careful to keep it out of the optical path, to help reduce points of entry for dust.

### Microscope platform preparation

We used a high-precision anodized-aluminum breadboard as the base to install all the microscope components, but it is not required. It can be substituted with an aluminum sheet of similar dimensions or thicker to support the weight of the microscope while providing sufficient strength at a light weight. To prepare the microscope platform:

• Cut a 37 mm hole out of the breadboard or microscope base according to the file titled BreadboardDrawing.pdf. It can either be milled or cut out using a 37 mm hole saw with the pilot bit centered on the breadboard hole at coordinates (6, 8) such that the origin is the front left corner of the breadboard.
• Debur the hole and clean any pieces of debris that may damage the optics.
• Attach rubber, vibration damping feet to one side of the posts and the breadboard to the other side.
• Place the microscope base into its intended location (e.g. cell culture incubator, final bench position).
• Install the optical rail at the edge furthest from the front of the breadboard as shown in the drawing titled MicroscopePlatformDrawing.pdf.
• Attach the rail carrier to the precision optical rail and use the thumb screw to fix it near the center line of the breadboard.
• Attach the T-slotted frame mounting foot for 1.5″ rails to the rail carrier using the T-framing fastener.

### Motor installation

To attach the motor to the fine hex adjuster on the Z-stage:

• Screw in the fine hex adjustor all the way, then unscrew it 3 turns in order to avoid damaging the motor during testing and allow the correct installation of the flexible shaft.
• Attach the motor to the motor bracket.
• Install the motor rail on the bottom side of the microscope breadboard 2 in. away from the fine hex adjustor (Fig. 1a, b) and Z-stage using the 3 corner brackets and their screws on any 3 of the 4 corners.
• Connect the fine hex adjustor and the motor using the flexible shaft.
• Attach the motor bracket to the motor rail using the T-frame fastener by sliding the base inside the motor rail and using the screws to fix them in place.
• Adjust the height of the motor to allow the flexible shaft to be straight without putting tension on it and keep the motor from touching the benchtop surface.

### Stage adapter fabrication

Using the aluminum bar and a mill, fabricate the adapters for the stage according to the AdapterForStage.pdf drawing provided as a supplemental file. Online services such as SendCutSend or its equivalent can also be used by submitting a scale drawing made in AutoCAD or Adobe Illustrator. This part can be substituted with a 3D printed one if not used with biological samples, as the layer lines of fused deposition modeling (FDM) 3D printed models make it difficult to keep the system sterile for use in a cell culture incubator. Another option is to 3D print the part using a material that can be autoclaved, such as polypropylene or polycarbonate.

### Illumination arm installation

The first installation of the LED does not need to be precise as it will be adjusted based on both the microscopy modalities required (e.g. brightfield vs. darkfield) and the centering procedure described in the validation and characterization section below.

**Fig. 3:**
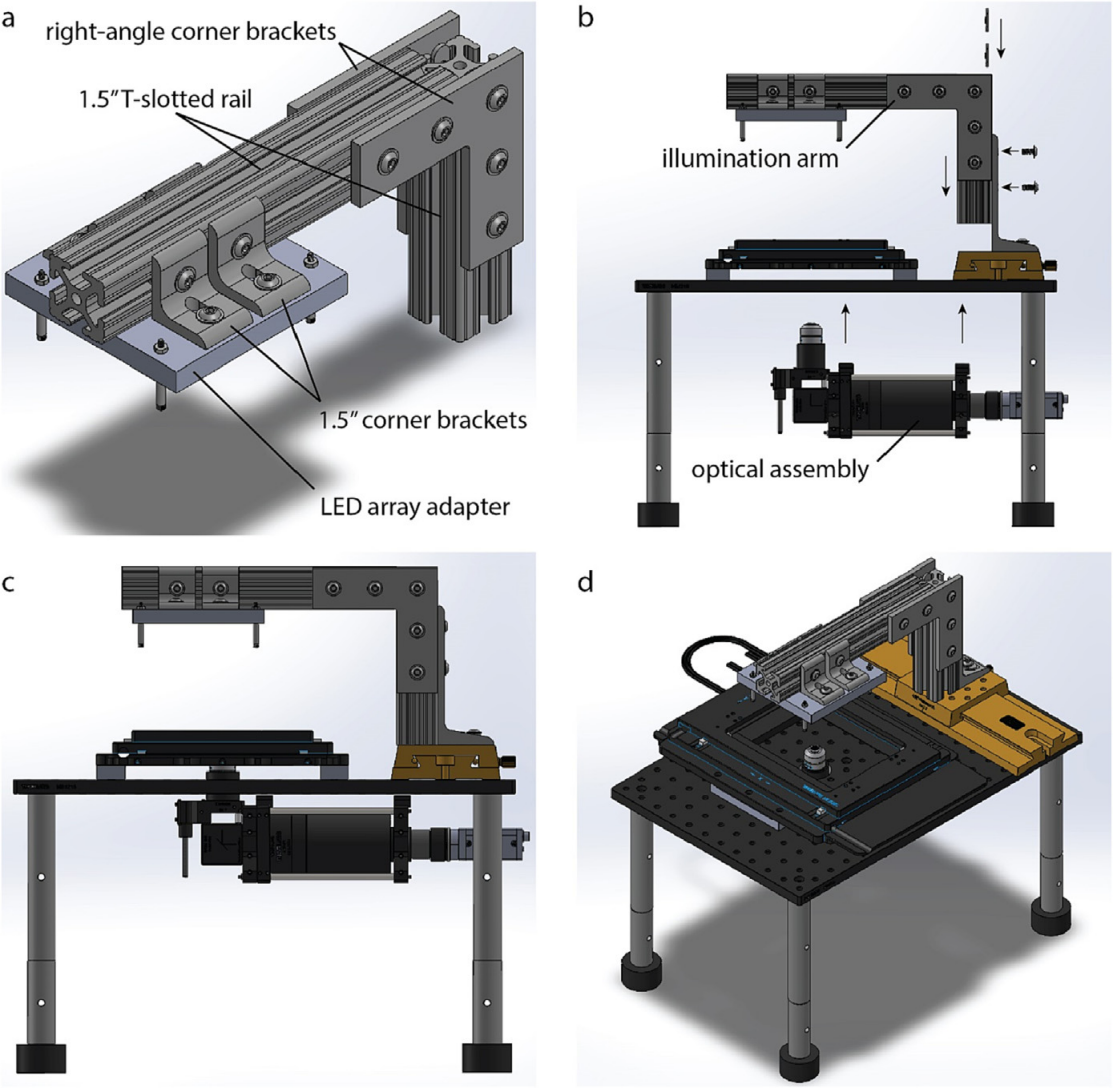
3D model of microscope assembly from individual subsystems. (a) Isometric view of Illumination arm (b) Diagram showing how illumination arm and optical assembly attach to the microscope platform. Arrows indicate direction of movement during installation (c) Side view of 3D model of assembled microscope. (d) Isometric view of 3D model of assembled microscope. Motor, motor rail, flexible shaft and LED array aren’t shown.
• Fabricate the LED array adapter according to the drawing titled LEDArrayAdapterDrawing.pdf. This can be done using a CNC mill or ordered using the drawing from SendCutSend.
• Attach 4 corner brackets to the aluminum LED array adapter.
• Attach the LED array adapter using LED screws, LED nuts, and unthreaded spacers. Use the T-framing fasteners and the 1.5″ corner brackets to interface the LED array adapter to the T-slotted rails. Slide the LED array adapter along the T-slotted rail to the place where the 4 panels of the LED array are approximately centered over the breadboard drilled hole then tighten the T-framing fasteners to fix the LED array in place.

• Using two right-angle corner brackets, attach the two T-slotted rails together using the T-framing fasteners, as shown in Fig. 3a.

### High-speed XY-stage installation

• Fabricate the stage adapters using the plans titled AdapterForStageDrawing.pdf. Alternatively this part can be ordered from SendCutSend.
• Attach the stage adapters as shown in the plans titled MicroscopePlatformDrawing.pdf using four ¼-20 screws.
• Attach the high-speed XY-stage to the adapters.
• Level the stage using a sensitive level (±1°) and shims placed between the stage and its adapter when the microscope is in its final location where it will be used for image acquisition.

### Wiring of the Arduino Metro

Make sure to use sufficiently long wires (approximately 30–50 cm) for wiring the Arduino Metro.

**Fig. 4:**
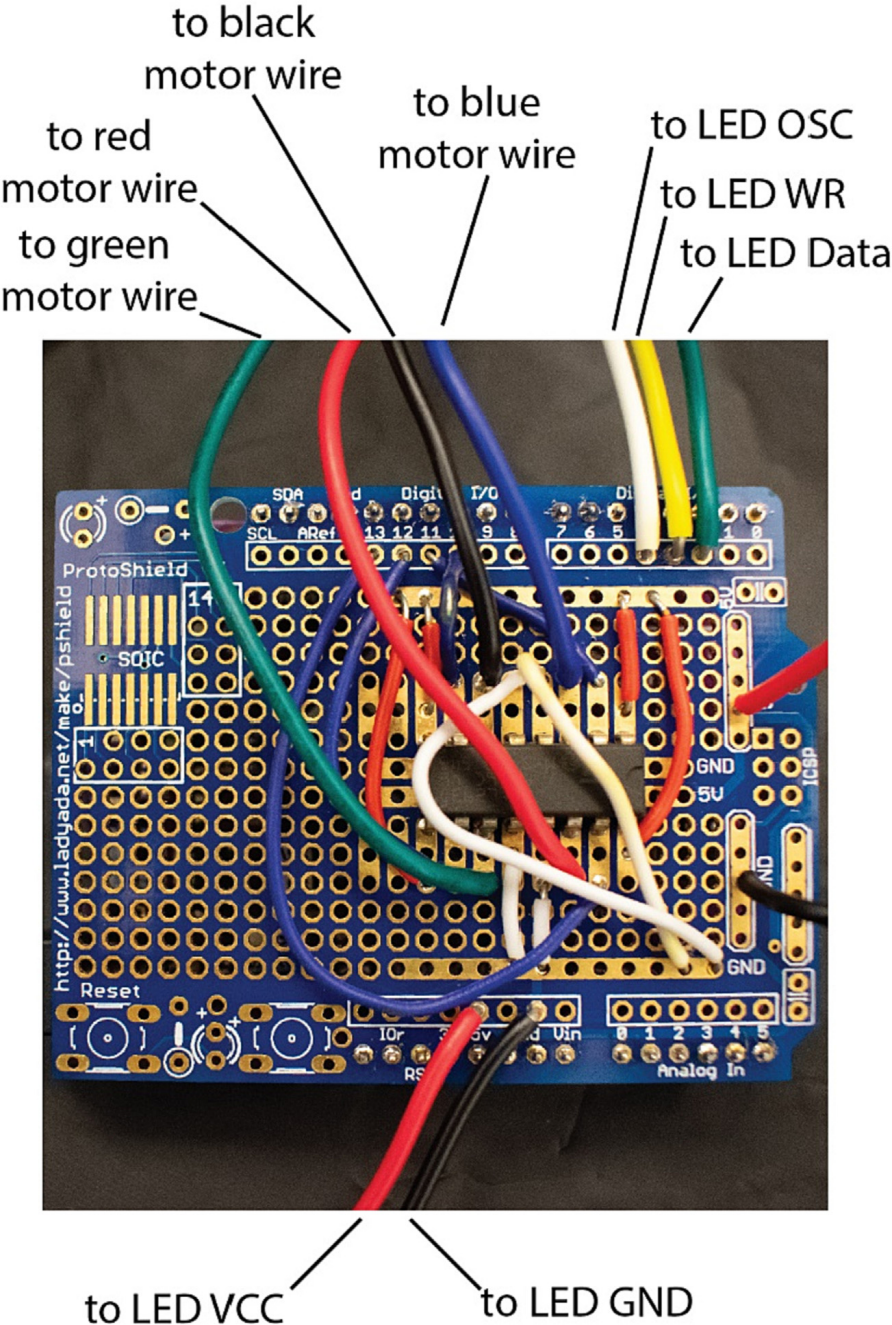
Wiring for LED array microscope. Photograph shows the LED array and Z-stage motor controller wired for connection to the Arduino using a proto shield.

**Fig. 5:**
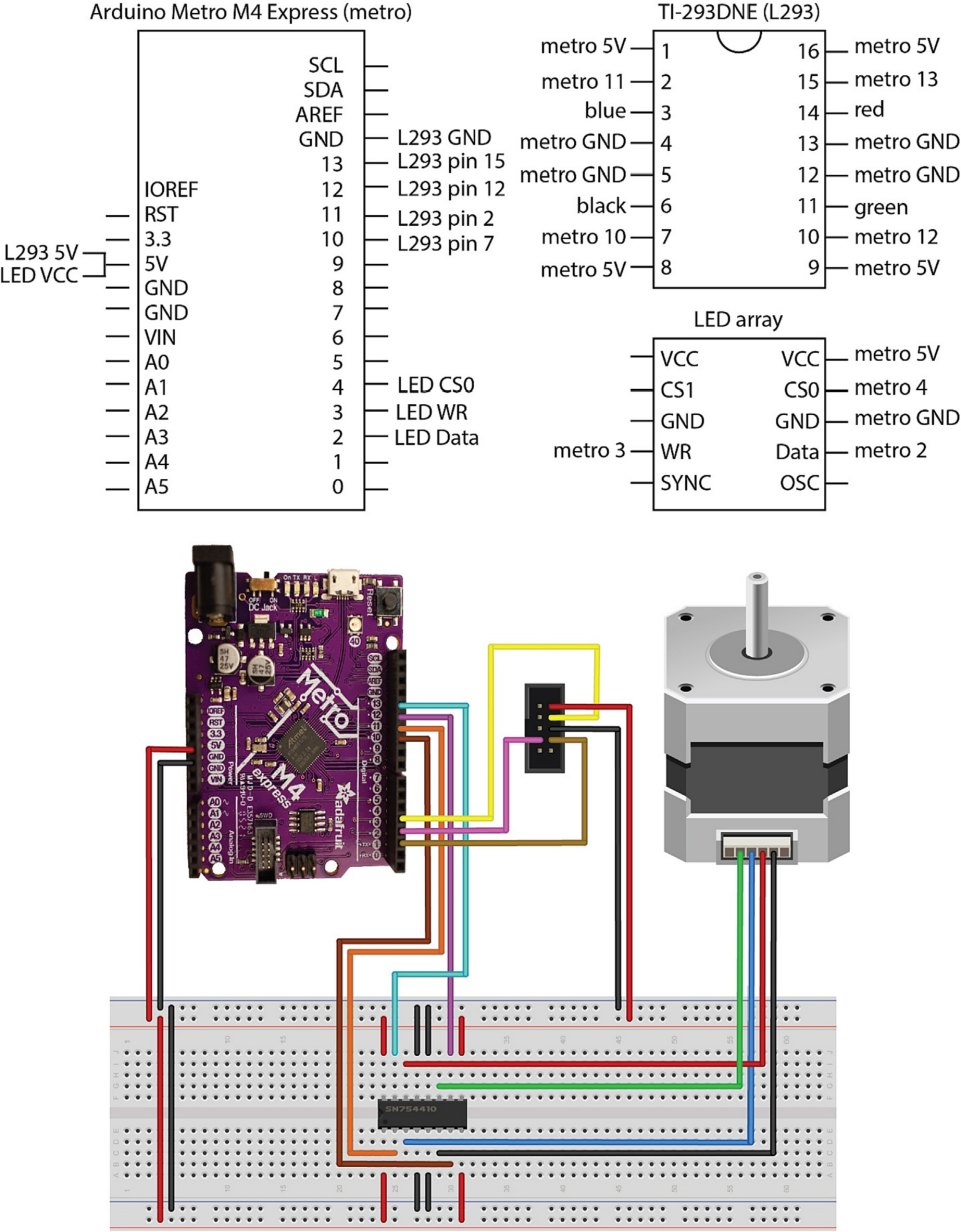
Wiring diagram for the microscope. Wiring diagram shows all pins for the Adafruit Metro M4 Express, TI-293DNE motor controller, and the LED array. Colors correspond to the wires shown in Fig. 4. (M4 image: Adafruit Fritzing library).
• Attach the proto shield to the Metro.
• Follow the instructions at this link: https://learn.adafruit.com/adafruit-metro-m4-express-featuring-atsamd51/setup to set up the Arduino IDE with the appropriate libraries to flash code to SAMD Arduino Boards. Alternatively, you can skip this step if you choose to use an Arduino Uno.
• Follow the instructions at this link: https://learn.adafruit.com/16x24-led-matrix/introduction to set up the Arduino IDE with the appropriate libraries to run the LED array.
• Connect the Metro to a computer using a micro-USB cable.
• Flash the provided code (MultiModalArduinoCode.ino) for the Arduino using the Arduino IDE.

• Follow the wiring diagram to wire the motor and LED array to the proto shield (Fig. 4 and Fig. 5).

### Final steps

• Connect the camera to the computer using the camera USB.
• Install the microscope objective onto the lens tube attached to the top of the Z-stage.

## Operation instructions

**Fig. 6:**
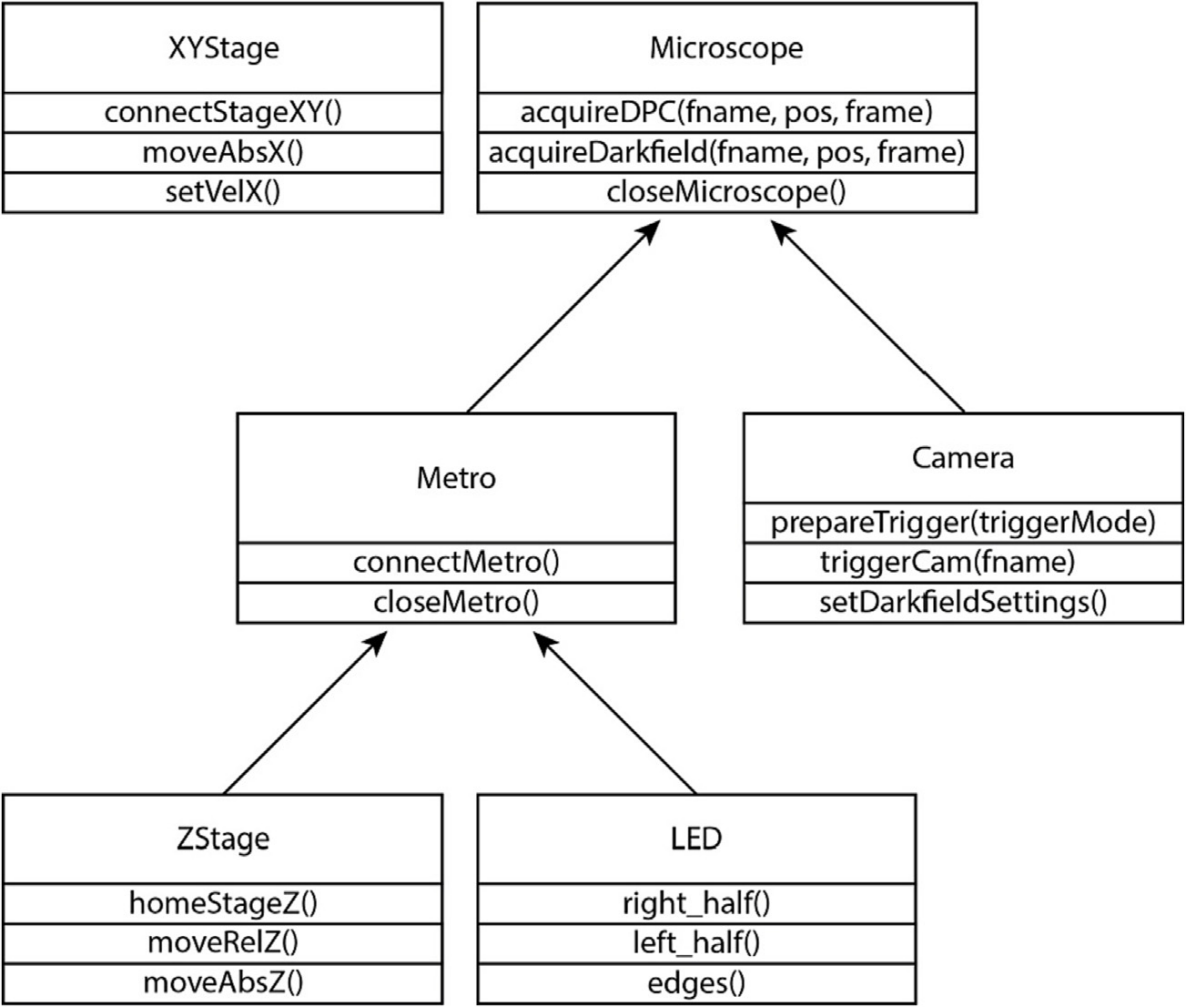
Flow chart showing hierarchy of inheritance. Metro class inherits the member variables and member functions of subclasses ZStage and LED. Ultimately all members of ZStage, LED, Metro, and Camera classes are inherited by the Microscope superclass. XYStage class is a standalone class that is called independently of the others. A subset of important member functions is shown under the class in which they are defined.

**Fig. 7:**
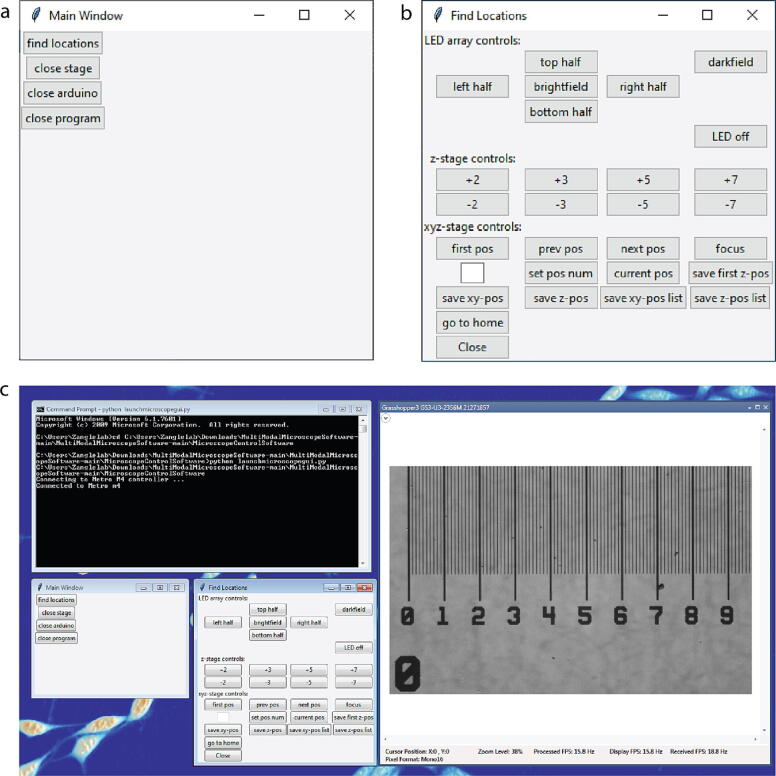
Screenshots of microscope GUI. (a) Main window when launching the GUI and (b) the Find Locations window that is used for identifying and focusing locations to image. (c) Desktop view with the GUI, command window to run Python commands, and live view using SpinView camera software.

Table 1 shows the essential functions for operating the microscope using the Python software package provided in the supplement (github.com/Zangle-Lab, 10.5281/zenodo.6841116). Each piece of hardware has its own Python class, making the software configurable in case alternative parts are used. If hardware from another manufacturer is used, changes to the class corresponding to this hardware will be required. However, if the same functions are used, the other classes will not be affected. For example, use of a camera from another manufacturer would require changes to the functions in the Camera class only. Inheritance is used based on the class hierarchy shown in Fig. 6 to allow for functions to interface the different pieces of hardware together. These functions can be called directly from the command line for direct control over the microscope. Alternatively, we included a graphical user interface to make it easy to connect to the microscope and control the basic functions, such as control over the LED array, focusing the microscope, saving positions, and exporting a position list (Fig. 7). Images can easily be acquired using the GUI that comes with the camera once the appropriate illumination setting is used. Furthermore, a routine called AcquireSnapshots.py can be used to visit saved positions and automatically acquire brightfield, darkfield, and the four asymmetric illumination images that can be used for phase retrieval with the included software [4], [5], [6], [7].

**Table 1:** Essential command line functions.

class	function name	inputs	outputs
Microscope	acquireDPC(froot, pos, frame)	froot: filepath to save location	none
		pos: position number	
		frame: frame number	
Microscope	acquireDarkfieldImage(froot, pos, frame)	froot: filepath to save location	none
		pos: position number	
		frame: frame number	
Microscope	closeMicroscope	none	none
Camera	prepareTrigger(triggerType)	triggerType = 0 for software trigger	none
		triggerType = 1 for hardware trigger	
Camera	triggerCam(filename)	filename: path and name of file to save	none
Camera	closeCamera()	none	none
Camera	changeExposureTime(exposure_time_to_set)	exposure_time_to_set: new exposure time to set	none
Metro	connectMetro(serial_port, baud_rate, read_timeout)	serial_port: 'COM#' for Arduino	none
		baud_rate: baud rate for serial connection	
		read_timeout: read timeout in seconds	
Metro	closeMetro()	none	none
ZStage	moveZ(steps)	steps: number of steps motor should move	none
ZStage	homeStageZ()	none	none
ZStage	moveRelZ(um)	um: distance to move in microns from current location	none
ZStage	returnToHome()	none	none
ZStage	moveAbsZ(um)	um: absolute z-position to move to	none
ZStage	focusHere(myWellNum)	myWellNum: location to focus on position list	none
ZStage	createPositionListZ()	none	none
ZStage	goToPositionZ(pos)	pos: position number to focus	none
ZStage	printPositionListZ()	none	none
ZStage	savePositionListZ()	none	none
ZStage	numPositions()	none	none
ZStage	savePositionZ()	none	none
ZStage	setWellNumZ(wellNum)	wellNum: location number to set as current location	none
ZStage	incrementWellNumZ()	none	none
ZStage	getPosZ()	none	none
LED	send(phrase)	phrase: command to send Arduino to illuminate custom pattern	none
LED	left_half()	none	none
LED	right_half()	none	none
LED	top_half()	none	none
LED	bottom_half()	none	none
LED	edges()	none	none
LED	full()	none	none
LED	small()	none	none
LED	off()	none	none
StageXY	createFocusLoc()	none	none
StageXY	createFirstWellCenter()	none	none
StageXY	createPositionListXY()	none	none
StageXY	connectX()	SerialNum: serial number for X-stage	none
		HWTYPE = 44 for BBD202	
StageXY	connectY()	SerialNum: serial number for Y-stage	none
		HWTYPE = 44 for BBD202	
StageXY	getVelX()	none	get x-velocity
StageXY	getVelY()	none	get y-velocity
StageXY	setVelX()	maxVel: maximum velocity desired in x-direction	none
StageXY	setVelY()	maxVel: maximum velocity desired in y-direction	none
StageXY	homeX()	none	none
StageXY	homeY()	none	none
StageXY	getPosX()	none	current x-position
StageXY	getPosY()	none	current y-position
StageXY	moveAbsX(um)	um: absolute x-position to go to	none
StageXY	moveAbsY(um)	um: absolute y-position to go to	none
StageXY	moveRelX(um)	um: distance to move in x-direction (in micrometers from current location	none
StageXY	moveRelY(um)	um: distance to move in y-direction (in micrometers from current location	none
StageXY	closeStageX()	none	none
StageXY	closeStageY()	none	none
StageXY	closeStageXY()	none	none
StageXY	goToPosition(pos)	pos: location in position list to move to	none
StageXY	printPositionList()	none	none
StageXY	numPositions()	none	none
StageXY	setWellNumXY(wellNum)	none	none

### Configuring Python environment to work with Grasshopper3 camera

• Install Python 3.6. This is the only version compatible with Spinnaker (Teledyne-FLIR)
• Download Spinnaker from the Teledyne-FLIR website and install both Spinnaker and SpinView (camera GUI)
• Install Pyspin using the command: $ python -m pip install pyspin

### Configuring Python software package to work with the Arduino and high-speed stage

To operate the microscope, a few of the default settings need to be modified. First, navigate to the folder containing the open-source software package written in Python, open the file Metro.py, and change the default value of the variable serial_port to the appropriate COM port for the Arduino on your computer, which can be identified using Device Manager. Next open the file StageXY.py; the first two functions are:

connectX(self, SerialNum = ##, HWTYPE = 44)

connectY(self, SerialNum = ##, HWTYPE = 44)

Change the variable SerialNum to be equal to the serial number on the back of the stage controller BBD202. There should be a different serial number for both the X and Y stages. This will allow the StageXY class to communicate with the high-speed stage.

### Navigating the microscope graphic user interface (GUI)

To start the GUI, turn on the high-speed XY-stage, and hold down the home button on the joystick for 5 s and release to home the stage. Navigate to the folder containing the provided Python software package and run the script LaunchMicroscopeGUI.py to make the main window appear (Fig. 7a). Press the button “find locations” to gain control over the microscope system. This will make a new window called “Find Locations” appear (Fig. 7b). At the top of the window are the controls for the LED array, allowing for various modes of illumination, including brightfield, darkfield, or asymmetric illumination, by selecting one of the buttons to illuminate from the top, left, bottom, or right halves of the LED array. There is also a button to turn the LED array off. In order to see the sample to focus and select positions you must be running the current version of the SpinView software distributed by Teledyne-FLIR (Fig. 7c).

### Using the *z*-stage to focus using the GUI

Underneath the controls for the LED array are the controls for the *z*-stage. The buttons allow for discrete movements of the *z*-stage up or down to focus the sample on the stage. If the sample is transparent (e.g. cells, microbeads, etc.), it is often easier to focus the sample using asymmetric illumination due to the lack of contrast when using standard brightfield.

### Creating an *x-y-z* position list

To create a position list for the coordinates to be imaged:

• Use the joystick to navigate and find the region of interest to be imaged.
• Once located, click the button “save xy-pos” to log the coordinates for this position.
• Then use the Z-stage motor controls to focus the sample in the field of view.
• Once the sample is in focus, click the button “save z-pos” to log the focus position of the objective lens. This will allow you to return to this position later and automatically refocus it.
• Additional positions may be logged by repeating steps 1–4 of this procedure.

### Reviewing saved positions

• Saved positions can be reviewed by clicking on the “first pos” button, returning you to the first position that you logged.
• The image may be out of focus. It can be focused automatically by clicking the button “focus” as long as a focus position was previously logged for this position.
• The “next pos” button will allow you to cycle through positions to review selected positions. You can go back to the previous position by clicking “prev pos.” At each position, click focus to automatically focus the location using the previously saved focus position.
• When finished, click the “save xy-pos list” button to save the *xy* locations to be imaged to a.csv file. Then click the “save z-pos list” button to save the focus positions to a file.

### Acquiring images

To acquire images:

• Press the “close button” to close the Find Locations window. This will return you to the Main Window.
• Press the button “close stage” to close the connection to the XY-stage.
• Press the button “close metro” to close the connection to the Arduino Metro M4 Express.
• Press the button “close” to close the GUI.
• Open the file “AcquireSnapshots.py” and set the variable froot to the file path where you would like to save the images.
• Set the variable framesPerHr to the number of frames you want to image at each saved location each hour. It will then automatically compute the amount of time to wait between each imaging cycle.
• Set the variable numFrames to the total number of frames to image at each location.
• Return to the command line and run the script “AcquireSnapshots.py” to cycle through each position and acquire four brightfield images with asymmetric illumination and a darkfield image. The frames for each location will be saved in a different folder named “pos#” such that # is the position number based on the order in which they were saved.
• To process the brightfield and phase images, open the file “DPC_main.m” in Matlab.
• Change the variable froot to the file path containing the folders named “pos#” such that # represents the location number for each imaged location.
• Set the variable numPos to the number of positions that were imaged.
• Set the variable numFrames to the number of frames imaged at each location.
• Set the variable fstart to be equal to a string that describes the experiment, usually “samplename_date_”. The position and frame number will be appended to the end of fstart to give each processed image a unique filename.
• Set the variable: rot_an = [90 180];
• Set the variable: reg2 = 1e-3;
• Click “run” to process the images. The images will be saved in froot, and each file will have a unique name. Each of the saved images will be saved in the following format: fstart_pos#_frame#.mat. Each of the saved.mat files contains two variables. Phase is the raw phase image computed from the four asymmetric images obtained from the microscope. BF is the brightfield image.

## Validation and characterization

### LED array centering

**Fig. 8:**
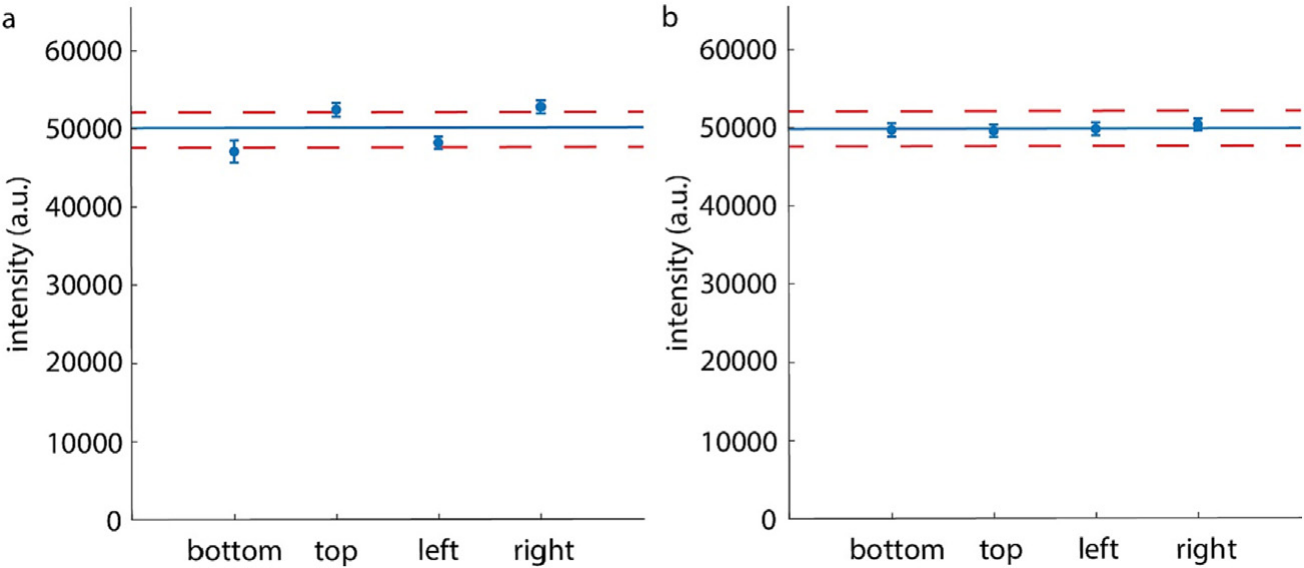
Centering the LED array: (a) Data showing LED array intensity when the LED array is uncentered. (b) Data showing the LED array intensity when the LED array is centered. Error bars show the standard deviation. Dark blue line shows the combined mean of all 4 halves of the LED array. Dashed lines show noise floor. (For interpretation of the references to colour in this figure legend, the reader is referred to the web version of this article.)

Custom built systems require rigorous characterization to validate proper function. The first important calibration step is to validate that the LED array is centered over the objective lens to ensure that the intensity of light incident on the camera is equal from each half of the LED array illuminated at a given time. This is especially important in the case of quantitative measurements because regardless of whether the measurement is reconstructed either in the spatial domain or Fourier domain, *a priori* knowledge of the geometry of microscope elements, such as the locations of individual LEDs on the array relative to the objective lens, is used for the reconstruction of the quantitative data [19]. When the LED array is uncentered, the intensity measured by the camera will be very different than when individual halves of the LED array are illuminated (Fig. 8a). However, when the LED array is centered properly, the mean intensity from all halves combined is within the noise floor of the mean intensity (Fig. 8b). To move the LED array, unscrew the 1.5″ corner brackets from the LED array adaptor or 1.5″ T-slotted rails to adjust the x-axis or y-axis respectively. The noise floor is the mean of an empty field of view without illumination +/- one standard deviation.

### Calibration of magnified pixel size

**Fig. 9:**
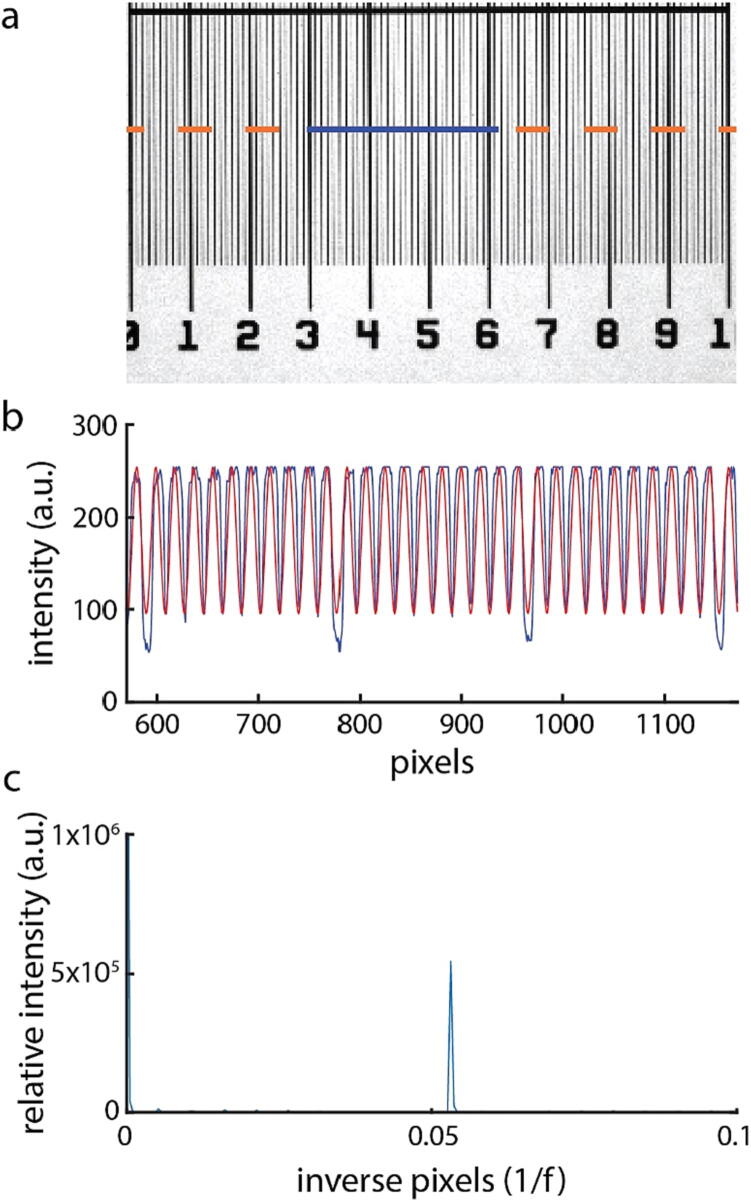
Calibration of magnified pixel size. (a) Image of a 1 mm transparent ruler. Small graduations are 10 µm apart and large graduations are 100 µm apart. The blue line in (a) is plotted in (b) as a blue curve. The red curve in (b) shows a sine wave at the spatial frequency identified by power spectral analysis. (c) The power spectrum of the blue curve shown in (b). (For interpretation of the references to colour in this figure legend, the reader is referred to the web version of this article.)

Proper calibration of pixel size is important for everything from adding a relevant scalebar to images as well as for proper computation of optical volume from QPI images. To calibrate the pixel size, we imaged a transparent ruler with 10 µm graduations (Fig. 9a). We then plotted a line through the image (Fig. 9b, blue curve) to show the raw image data. We computed the power spectrum of this curve to determine how far apart the graduations are (in pixels) based on the spatial frequency of intensity changes (Fig. 9c). To validate the distance between graduations, we plot a sine wave of the same frequency and find that the frequency of the sine wave matches the frequency of the graduations from the raw image data (Fig. 9b, red). This sine wave also serves as a quick visual check that the power spectral analysis returned the correct result, without aliasing, or other artifacts.

### Calibration of image acquisition and reconstruction parameters

**Fig. 10:**
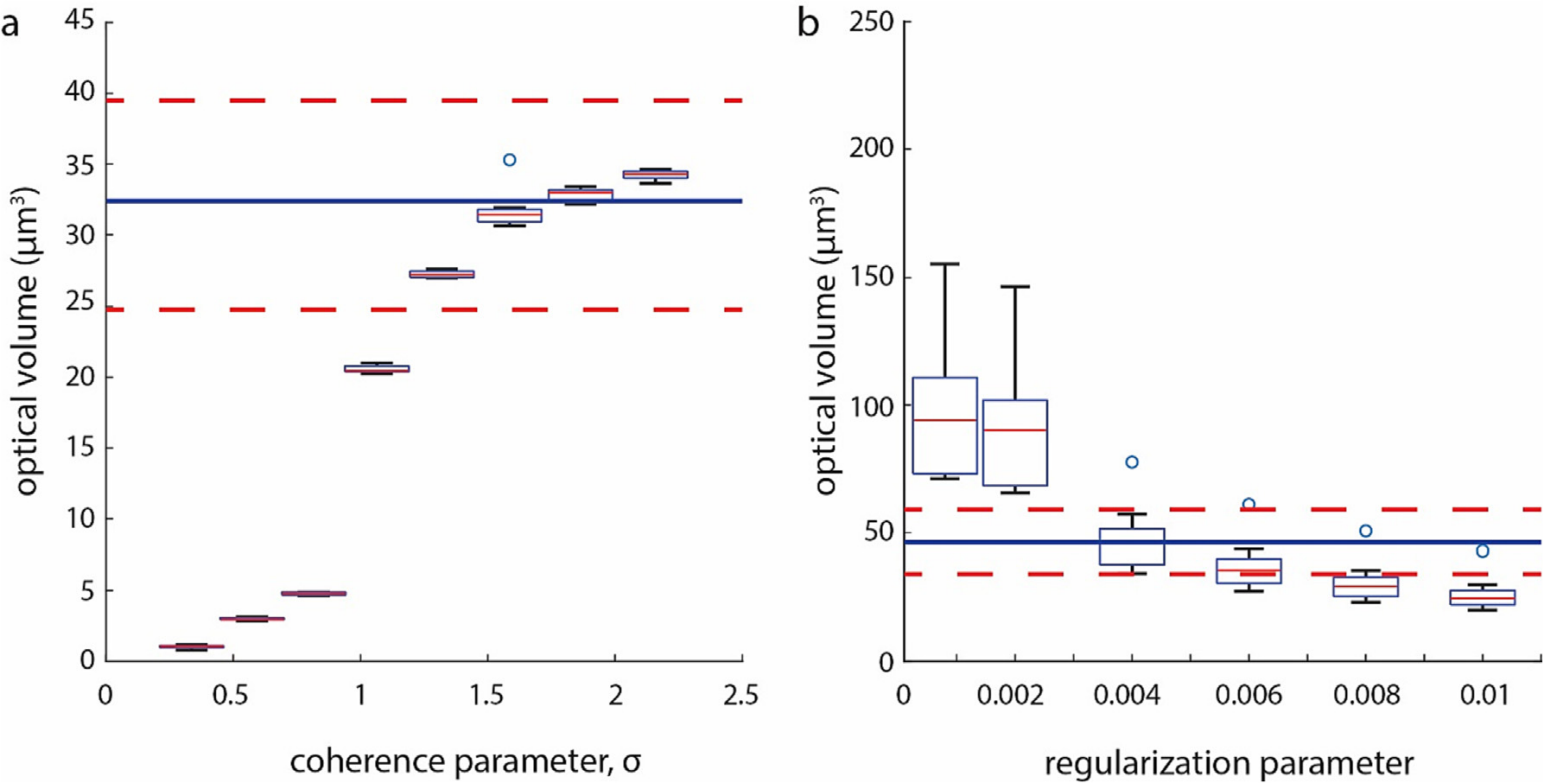
Calibration of coherence parameter and regularization parameters. (a) Plot of optical volume vs. coherence parameter. Regularization parameter was held fixed at 0.004. (b) Plot of optical volume vs. regularization parameter. Here, coherence parameter was help fixed at 2. In both plots, the dark blue line shows the theoretically predicted mean based on the difference in reported difference in refractive index and the size of the beads. Dashed red line shows the expected standard deviation based on the difference in sizes of the individual beads. (For interpretation of the references to colour in this figure legend, the reader is referred to the web version of this article.)

An important consideration when building an LED array microscope is the size of the LED array and its distance from the sample. This can be summarized by the coherence parameter [6] of the system, which is defined in Eq. (1). We characterize the optical volume of polystyrene microbeads as a function of *σ* and found that the larger value of *σ*, the greater accuracy of the results. At a maximum tested value of *σ* = 2.2, we found that the measured optical volume was 38.7 µm^3^, about 5 % greater than the expected value of 36.9 µm^3^ and at *σ* = 1.9, the measured optical volume was 2 % lower than the expected value (Fig. 10a). More generally we found that for *σ* ≥ 1.5 the accuracy was with 5 % of the expected optical volume for the beads. Below a coherence parameter of 1.5 the accuracy of the optical volume measurements dropped dramatically, indicating that 1.5 is the minimum value of the coherence parameter that can be used to obtain quantitative phase measurements using this system.

Another key parameter for obtaining quantitative phase measurements is the regularization parameter used for the phase retrieval algorithm [6]. Once we identified that a higher coherence parameter is better for obtaining high fidelity phase measurements, we fixed our coherence parameter to *σ* = 2.1 to obtain the best contrast [6] while calibrating the regularization parameter used for phase reconstruction. While higher sigma is necessary for good contrast [6], the regularization parameter is system dependent and must be tuned based on the signal-to-noise ratio of the system. To do this we obtained a set of images using asymmetric illumination as an input for the phase retrieval algorithm, and we recomputed the phase images using a range of relevant regularization parameters. Unlike the coherence parameter, where we found that *σ* ≥ 1.5 was sufficient for phase reconstruction, there was a single best regularization parameter that balanced low noise measurements with the optical volume of polystyrene beads used as phase calibration standards (Fig. 10b).

### Temporal characterization of QPI

**Fig. 11:**
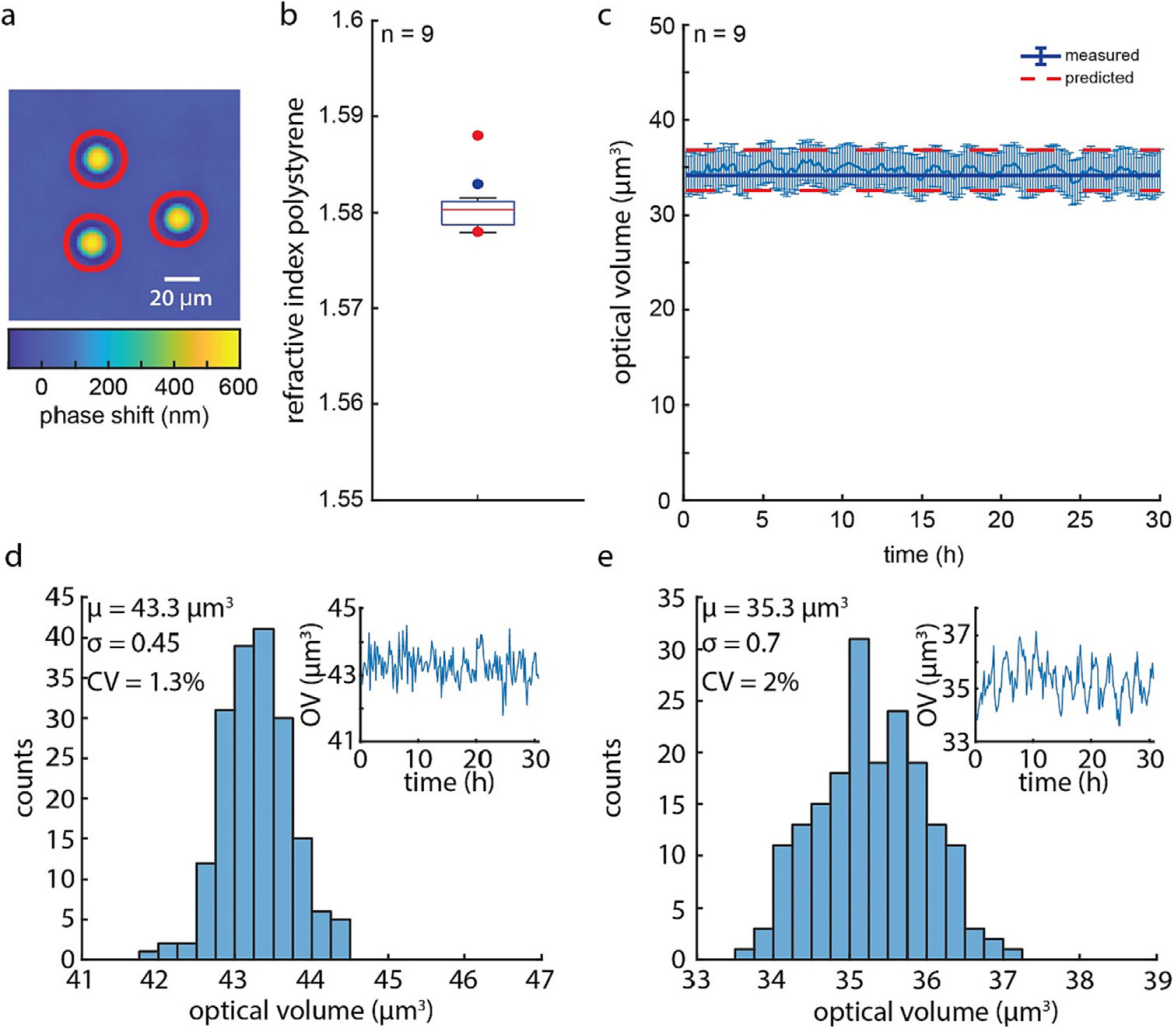
Validation of QPI by measuring the optical properties of known materials. (a) The optical path length through three polystyrene microbeads with segmentation outlined used for computing the optical volume of the beads shown in red. (b) Boxplot of the difference in refractive index between the polystyrene beads and NOA73. Blue dot represents the expected difference in refractive index and the red dots show the expected error based on the uncertainty in the measured optical properties of the two materials. (c) Optical volume of 9 microbeads measured over a 30 h duration. Dark blue line shows the expected mean optical volume, dashed red lines represent the expected standard deviation based on the different sizes of the beads. Error bars represent the standard deviation of the measurement. (d-e) Histograms from two beads used to compute the curve in (c) showing all the measurements made for each of the two beads throughout the 30 h duration. Inset shows the individual traces of optical volume measurements for the two beads. (For interpretation of the references to colour in this figure legend, the reader is referred to the web version of this article.)

We validated the quantitative phase measurements of this system by measuring the optical path length through polystyrene microbeads embedded in an NOA73 polymer (Fig. 11a). The optical path length for a material of uniform refractive index, such as a polystyrene bead, can be found as: OPL = *t* Δ*n*, where *t* is the thickness of the sample at a given point, and Δ*n* is the difference in refractive index between the sample material and the material in which it is embedded. Polystyrene is a commonly used material that has known optical properties, such as its refractive index (*n* = 1.583) [20], and previous work has used them as an inexpensive calibration standard for QPI [15], [21], [22], [23]. NOA73 (*n* = 1.56) is another inexpensive material that is readily available and has a similar refractive index to polystyrene beads. This close match in refractive index makes samples with this construction a reliable phase standard as they do not introduce phase wrapping errors [24], [25] that could be present if the OPL exceeded one half the wavelength of light used for imaging. We found that the measured refractive index of the microbeads embedded in NOA73 was 1.58, which is within 0.2 % of the expected value of 1.583 (Fig. 11b). We also quantified the optical volume of microbeads over a 30 h duration, which had a mean value of 34.6 µm^3^, which is 1.4 % greater than the theoretical value of 34.1 µm^3^ (Fig. 11c). Histograms of optical volume measurements of two of the beads shown in (Fig. 11d, e) show only small deviations from the mean value as measured by the temporal coefficient of variation of 1.8 % (Fig. 11d, e).

### Characterization of darkfield

**Fig. 12:**
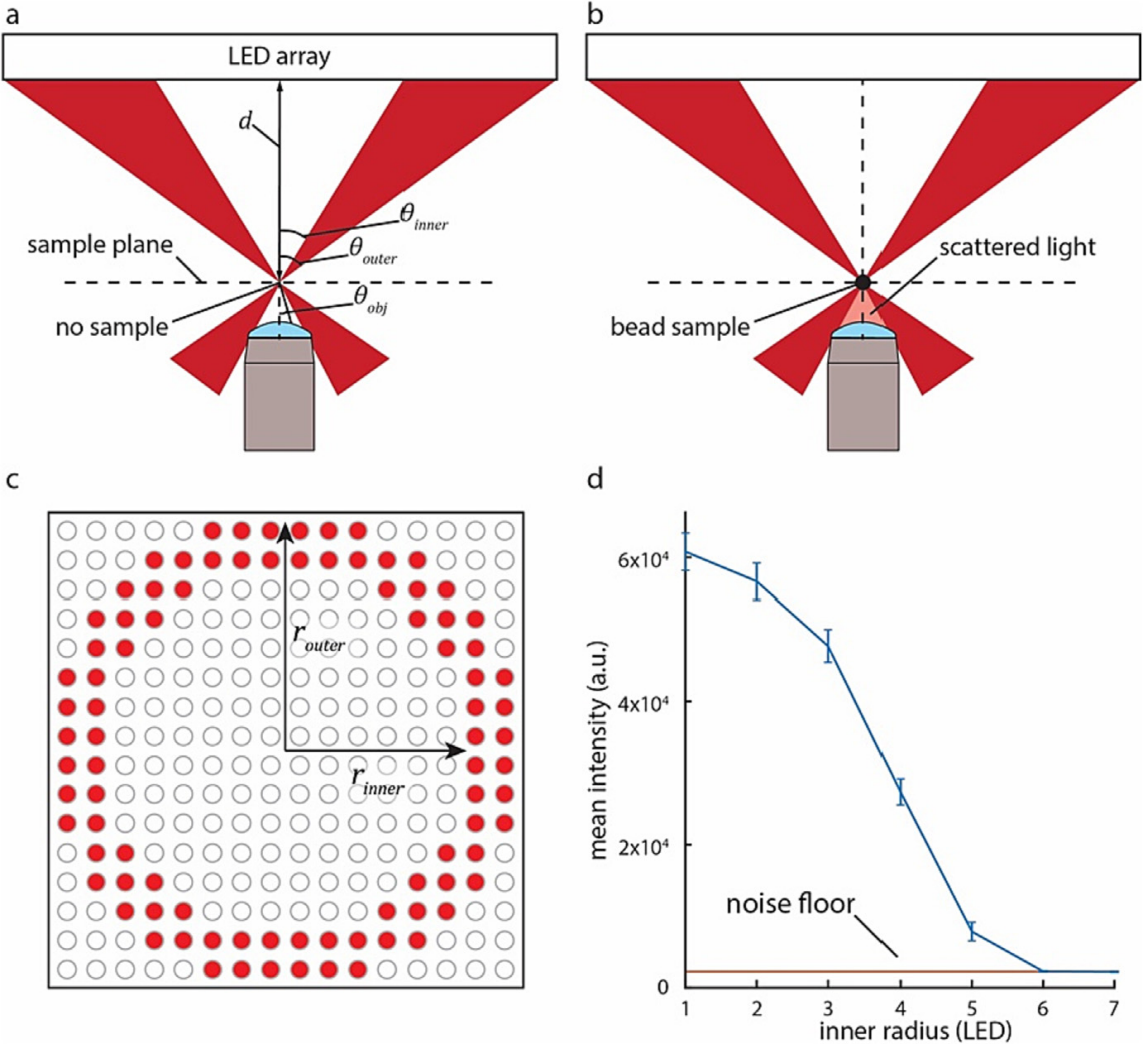
Darkfield description and characterization of illumination pattern. (a) In darkfield microscopy, when no sample is present, no light is scattered towards the objective resulting in no light being collected by the lens. (b) Light is only collected by the objective lens when it is scattered by a sample such as a microbead. (c) Darkfield illumination is annular and, therefore, characterized by both an inner and outer numerical aperture, here characterized by the radii of LED illumination on the LED light panel. (d) Plot of mean intensity as a function of the inner radius (measured in number of LEDs). Error bars show standard deviation, and the red line represents the noise floor when the LEDs are turned off. (For interpretation of the references to colour in this figure legend, the reader is referred to the web version of this article.)

Darkfield imaging measures scattered light by illuminating the sample from outside the numerical aperture of the objective lens. In the absence of a sample, the light used for darkfield imaging, therefore, cannot be collected by the objective lens (Fig. 12a). In the presence of a sample, however, light scattered by the sample is collected resulting in an image with a dark background with the sample having an intensity greater than the background (Fig. 12b). An important characterization step when constructing a darkfield microscope is to validate that the illumination is not reaching the objective lens other than when it is scattered by the sample, while also having enough illumination incident on the sample that enough is scattered to produce contrast at high signal to noise ratio. The darkfield illumination pattern can be characterized by both the inner and outer numerical aperture of illumination which can be calculated using the following equation:

(2) $$\mathrm{NA}=\sin\left(\theta \right)=\frac{r}{d}$$

where *θ* is the half angle of illumination, and *r* is the radius of the illumination pattern at distance *d* from the sample plane (Fig. 12c).

To find the correct illumination pattern, we used an outer numerical aperture of illumination of 0.51 (*σ_outer_* = 2.2) corresponding to using all 8 LEDs up to the edge of the 68 mm wide LED array (*r* = 32.5 mm) placed at *d* = 110 mm away from the sample for maximum illumination intensity. The NA of the objective is 0.25, therefore we expected that an inner numerical aperture greater than 0.25 (*σ* = 1, or an inner radius of 4 LEDs) would result in darkfield illumination. This corresponds to an inner radius of 4 LEDs with NA = 0.25. In practice, however, we found that an inner radius of 6 LEDs (NA_inner_ = 0.39, *σ_inner_* = 1.56) is required to achieve a completely dark background (Fig. 12d).

### Darkfield signal to noise ratio

To calculate the signal to noise ratio (SNR) for darkfield, we used the same beads sample used for phase validation. From these images, we estimate SNR as:

(3) $$\mathrm{SNR}=\frac{\mu_{\mathrm{bead}}-\mu_{\mathrm{empty}}}{\sigma_{\mathrm{empty}}}$$

**Fig. 13:**
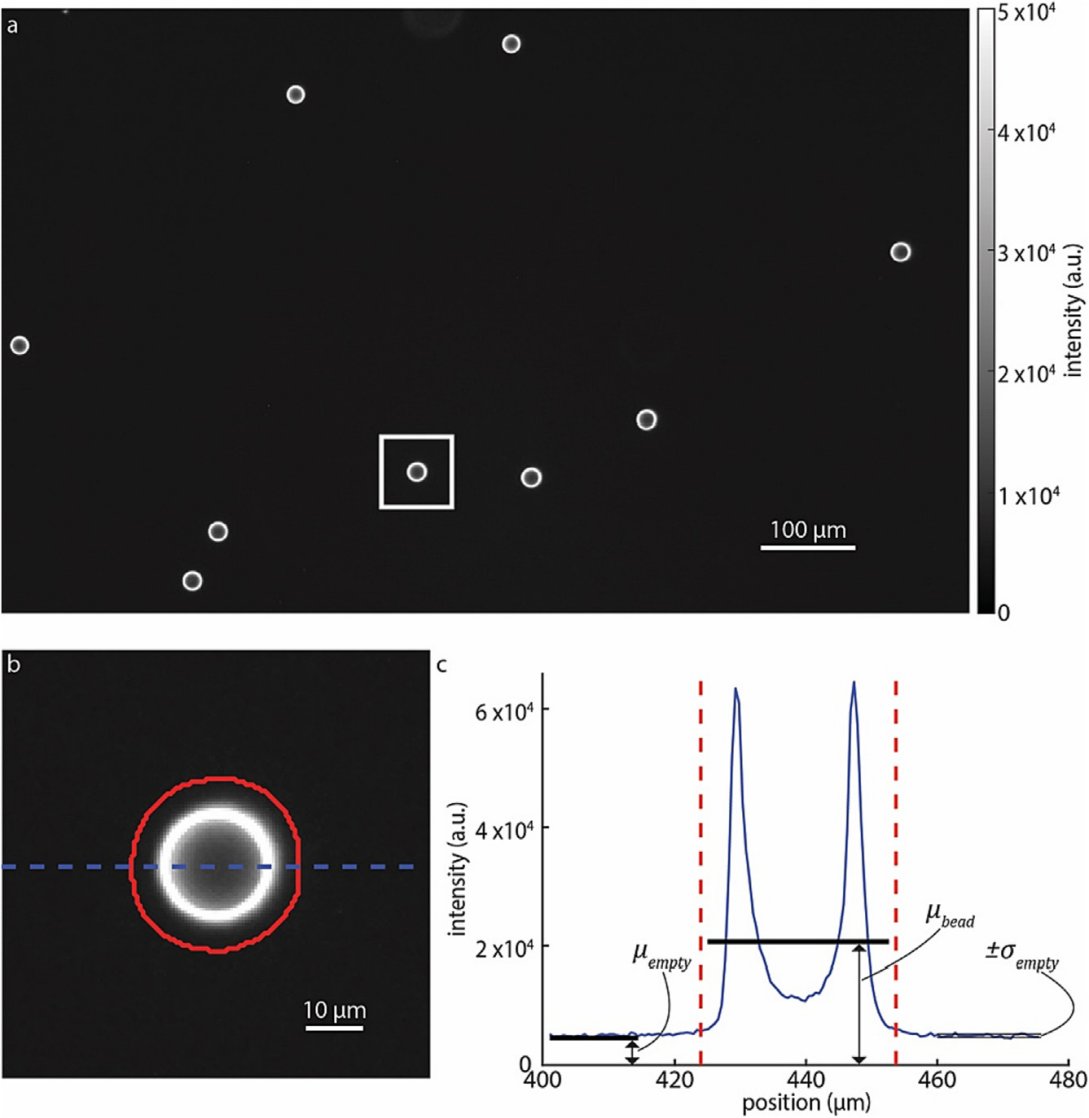
Signal to noise ratio of darkfield imaging of beads (a) Darkfield image of beads taken at exposure time of 220 ms with a single bead outline with a white rectangle. (b) Magnified view of bead outline in (a) with segmentation boundaries shown in red. The dashed blue line in (b) is shown as solid blue line in (c) with $\mu_{\mathrm{bead}}$, $\mu_{\mathrm{empty}}$, and $\sigma_{\mathrm{empty}}$ from Eq. (3) indicated. (For interpretation of the references to colour in this figure legend, the reader is referred to the web version of this article.)

Here, $\mu_{\mathrm{bead}}$ is the mean intensity of the bead after subtracting the mean intensity of the empty space in the image, $\mu_{\mathrm{empty}}$ divided by $\sigma_{\mathrm{empty}}$, the standard deviation of intensity in the empty space in the image. SNR, therefore, defines the ability of the system to detect the difference between measurement and random noise in the background. SNR is dependent on illumination intensity and pattern, camera settings (exposure time and gain), and type of sample and its properties. Using this procedure, we estimate an SNR of 50±7 (Fig. 13).

### Multimodal imaging of live cells

**Fig. 14:**
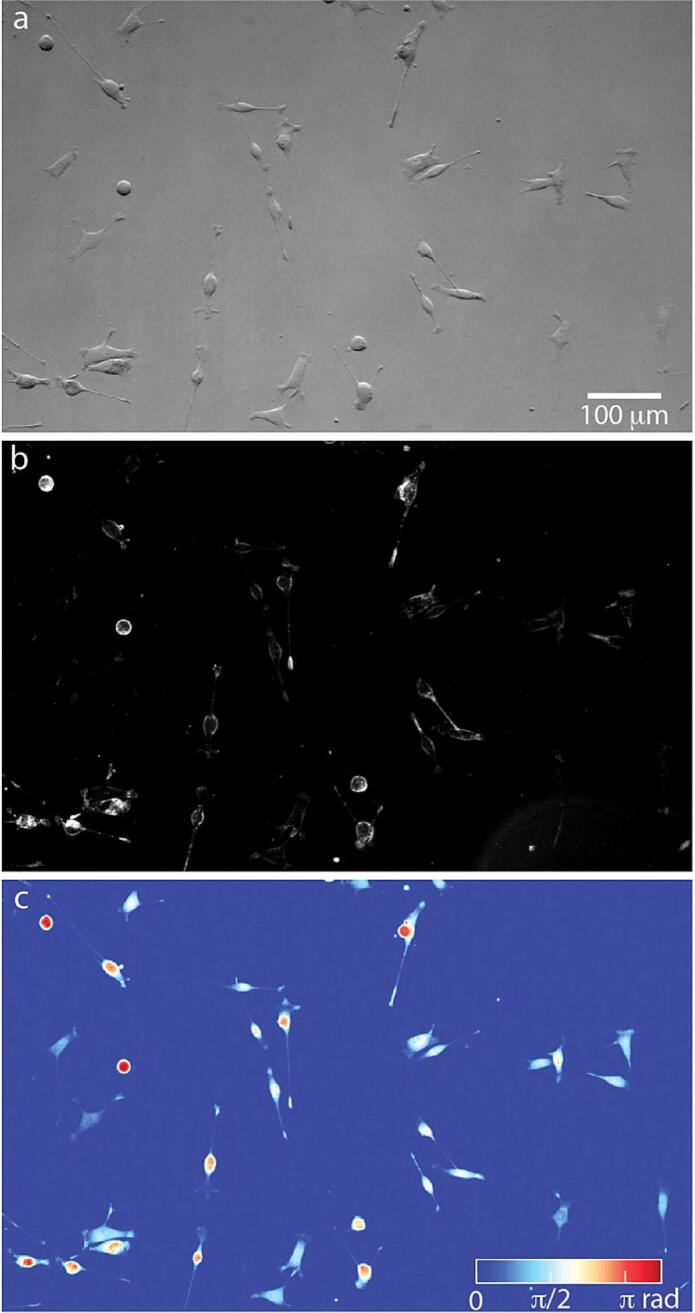
Sample images taken with multimodal LED array microscope. (a) Brightfield image taken with top half semi-circle illumination of MTG-021 PDX melanoma cells. (b) Darkfield image and (c) quantitative phase image of the same field of view as pal (a). Colormap in (c) shows phase shift with dark blue being background, and dark red a larger phase shift, such as that through the center of the round, mitotic cells shown in this image. (For interpretation of the references to colour in this figure legend, the reader is referred to the web version of this article.)

The entire microscope assembly is sized to fit within a standard cell culture incubator for live cell imaging. Although many of the microscope parts are not certified for operation in a high humidity (95 %) environment, we have not noted a degradation in performance of the system, including the camera and XY stage, over 3 device-years of operation. As a demonstration, MTG-021 melanoma cells established from a patient derived xenograft (PDX) were cultured in AM-3 media and plated at 3.4x10^3^ cells/cm^2^ prior to imaging with the multimodal microscope. AM-3 media consists of 80 % MCDB153 (Sigma-Aldrich, USA), 20 % L-15 medium (Gibco, USA), 2.5 % FBS (Denville Scientific, USA), 0.2 % bovine pituitary extract (Gibco, USA), 10 ng/ml insulin-like growth factor, 5 ng/ml EGF (Sigma-Aldrich, USA), 5 µg/mL transferrin, 3 ng/ml BFGF, 3 µg/mL heparin, 0.18 µg/mL hydrocortisone, 1.68 mM calcium chloride, and 10 nM endothelin-1. Brightfield imaging with one of the DPC half-illumination patterns highlights gradients in optical path length, enabling clear visualization of cells (Fig. 14a). Darkfield imaging produced clear images of cell boundaries, as well as visible intracellular puncta (Fig. 14b). Finally, QPI data reconstructed from the 4 quadrant DPC images quantifies the phase shift of light as it passes through the cells [4], [5]. These data can be used to quantify cell mass or the change in cell mass over time that occurs during growth [11].

**Fig. 15:**
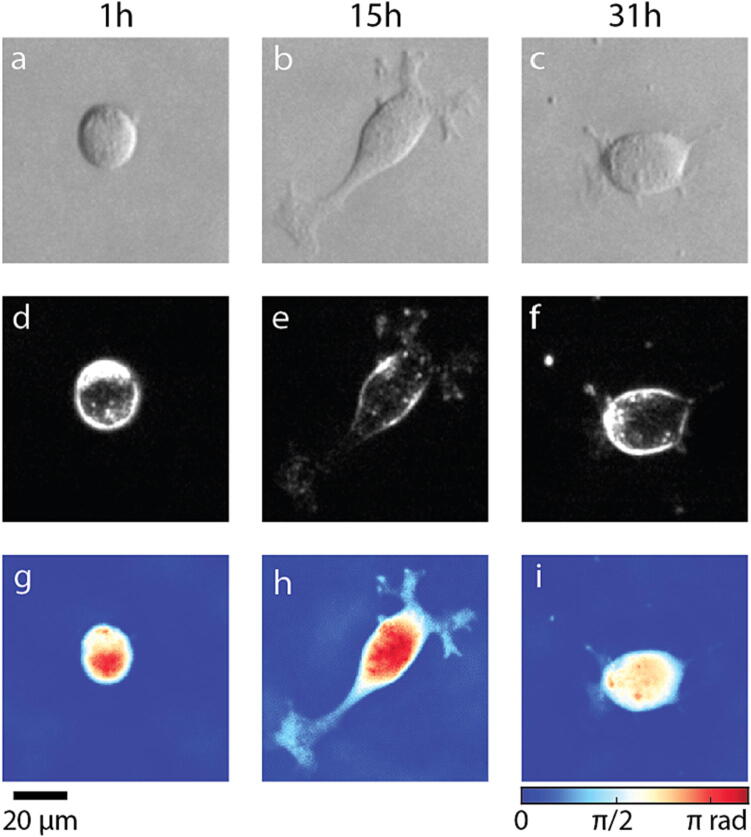
Close up images of a single cell over time. (a-c) Brightfield images of MTG-021 PDX melanoma cells taken with right half semi-circle illumination. (d-f) Darkfield images and (g-i) QPI data of the same field of view.

As an example of multimodal imaging, brightfield, darkfield, and QPI data of the same cell over time reveals distinct changes in the cell (Fig. 15). Brightfield imaging is the simplest and quickest of the 3 modalities requiring a single image with short exposure time (50 ms) and no post-processing (Fig. 15a-c). Brightfield is used to choose positions and set focus, and also reveals cell morphology. Darkfield imaging highlights cell edges and puncta (Fig. 15d-f), but with the requirement of relatively long exposure time (200 ms). Finally, QPI shows how cells grow over time (Fig. 15g-i). An operator can use phase images such as these to extract cell area, which in this case changed from 1,000 µm^2^ to 2,675 µm^2^ to 2,063 µm^2^ (1, 15, and 31 h, respectively). At the same time, the cell grew from 432 pg to 592 pg from 1 to 15 h, then lost mass by the 31 h timepoint to a final mass of 532 pg, indicating the possible onset of cell death.

## Conclusion

In summary, we demonstrated how to build, validate, and optimize a multimodal microscope that is research capable and versatile in design. Additionally, our validation protocol covers the needed aspects of quantitative validation for both quantitative phase and darkfield measurements. The resulting microscope is modular and can be extended, e.g. with the addition of illumination and a dichroic filter cube for fluorescence.

This system performs well in comparison to commercial systems, either in published work or within our own lab. Optical volume and refractive index measurements of polystyrene beads with this system agree with what we expect from known material properties (Fig. 11) as well as previous characterization of commercial systems [26], [27], [28], [29]. In previous work we have shown that measurements of cell mass and growth performed with a system based on these plans agree with commercial systems as well as independent measurements of cell growth [11]. Finally, the measured precision of ∼1–2 % is comparable to commercial systems [28]. Overall, this suggests good performance from this open-source design, suitable for a variety of image acquisition tasks.

Open hardware microscope designs tend to be purpose built for specific imaging modalities, including but not limited to fluorescence [30], brightfield [17], [31], darkfield, and light sheet imaging [32]. Although this specificity in design lowers the overall cost and complexity, it limits the application of these systems to different modalities or use cases. One possible solution is to have an open-source modular standardized system that easily allows operators to swap different parts for others without the need to completely build a new system with a new operating software. We tried to employ such philosophy in our design where an operator can swap parts such as the camera with the only required change on the software side being a change in a single class. More integrated development of hardware and software to expand such uses is needed, however.

More than half of the cost for the system presented here is the XY-stage and its controller. This XY-stage has remarkable speed, precision and accuracy, as required for some cases, such as drug screening [8]. However, there are multiple open hardware designs that can deliver accuracy in the micrometer range for a significantly reduced price [16], [17], [18], [30], [33]. Most of these open-source designs are made for biological applications and allow for smaller scale experiments to be performed. Some even have a small incubator or an enclosure integrated into the design to further lower the cost and required equipment [17], [18], [31], [34]. Additionally, as many components are optomechanical, open-source 3D printed alternatives could further lower the cost and overall weight of the system, resulting in lower required stiffness and cost for the support hardware [34], [35]. Therefore, in the spirit of open hardware, we consider this design as base for improvement and modifications for specific use cases.

## Ethics statements

The work presented in this study did not require the use of any human or animal subjects.

## CRediT authorship contribution statement

**Tarek E. Moustafa:** Conceptualization, Methodology, Software, Data curation, Formal analysis, Investigation, Validation, Visualization, Writing – original draft. **Edward R. Polanco:** Conceptualization, Methodology, Software, Data curation, Formal analysis, Investigation, Visualization, Writing – original draft. **Rachel L. Belote:** Methodology, Writing – review & editing. **Robert L. Judson-Torres:** Funding acquisition, Writing – review & editing. **Thomas A. Zangle:** Supervision, Conceptualization, Funding acquisition, Methodology, Project administration, Resources, Writing – review & editing.

## Declaration of Competing Interest

The authors declare that they have no known competing financial interests or personal relationships that could have appeared to influence the work reported in this paper.
